# Retinal Inflammation and Reactive Müller Cells: Neurotrophins’ Release and Neuroprotective Strategies

**DOI:** 10.3390/biology13121030

**Published:** 2024-12-09

**Authors:** Bijorn Omar Balzamino, Andrea Cacciamani, Lucia Dinice, Michela Cecere, Francesca Romana Pesci, Guido Ripandelli, Alessandra Micera

**Affiliations:** 1 Research and Development Laboratory for Biochemical, Molecular and Cellular Applications in Ophthalmological Science, IRCCS-Fondazione Bietti, via di Santo Stefano Rotondo 6, 00184 Rome, Italy; bijorn.balzamino@fondazionebietti.it (B.O.B.); lucia.dinice@fondazionebietti.it (L.D.); 2 Surgical Retina Research Unit, IRCCS-Fondazione Bietti, via di Santo Stefano Rotondo 6, 00184 Rome, Italy; andrea_cacciamani@hotmail.com (A.C.); michela.cecere@fondazionebietti.it (M.C.); francesca.pesci@fondazionebietti.it (F.R.P.); guido.ripandelli@fondazionebietti.it (G.R.)

**Keywords:** retinal disorders, Müller cells, aging, NGF, precision medicine

## Abstract

Millions of people worldwide suffer from retinal disorders. Retinal diseases require prompt attention to restore function or reduce progressive impairments. Genetics, epigenetics, life-styling/quality and external environmental factors may contribute to developing retinal diseases. In the physiological retina, some glial cell types sustain neuron activities by guaranteeing ion homeostasis and allowing effective interaction in synaptic transmission. Upon insults, glial cells interact with neuronal and the other non-neuronal retinal cells, at least in part counteracting the biomolecular changes that may trigger retinal complications and vision loss. Several epigenetic and oxidative stress mechanisms are quickly activated to release factors that in concert with growth, fibrogenic and angiogenic factors can influence the overall microenvironment and cell-to-cell response. Reactive Müller cells participate by secreting neurotrophic/growth/angiogenic factors, cytokines/chemokines, cytotoxic/stress molecules and neurogenic inflammation peptides. Any attempt to maintain/restore the physiological condition can be interrupted by perpetuating insults, vascular dysfunction and neurodegeneration. Herein, we critically revise the current knowledge on the cell-to-cell and cell-to-mediator interplay between Müller cells, astrocytes and microglia, with respect to pro-con modulators and neuroprotective/detrimental activities, as observed by using experimental models or analyzing ocular fluids, altogether contributing a new point of view to the field of research on precision medicine.

## Simple Summary

Retinal diseases require prompt attention to restore function or reduce progressive impairments. In the physiological retina, some glial cell types sustain neuron activities by guaranteeing ion homeostasis and allowing effective interaction in synaptic transmission. Several epigenetic and oxidative stress mechanisms are quickly activated to release factors that in concert with growth, fibrogenic and angiogenic factors can influence the overall microenvironment and cell-to-cell response. Reactive Müller cells participate by secreting neurotrophic/growth/angiogenic factors, cytokines/chemokines, cytotoxic/stress molecules and neurogenic inflammation peptides.

## 1. Introduction

**Figure 1:**
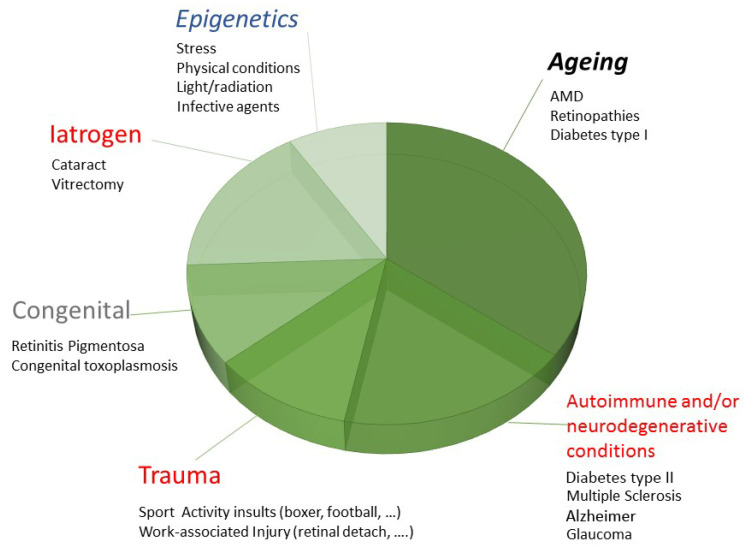
Overview of factors contributing to retinal disorders. The pie chart depicts the main retinal-insult-associated sources, including aging, epigenetics, genetic background and social stressors able to influence or produce insults that directly (mediator release) or indirectly (mediator release from other injured areas) offend the retinal structure and microenvironment.

Neurons, glia and vascular cells interact smoothly to maintain retinal homeostasis in the adult retina [1]. Several types of endogenous/exogenous (trauma, injury, autoimmune states, infections, even accumulation of extracellular toxins and aging) insults can trigger gliosis to protect the retinal network from impairments, which is achieved via different cell-to-cell and cell-to-mediator activities [1,2]. A number of retinal diseases, including diabetic retinopathy, age-related macular degeneration, retinal detachment, glaucoma-associated optic nerve disorders and retinal disorders, are characterized by a variable degree of inflammation, which plays an important role in diabetes-mediated retinal damage (Figure 1) [3,4]. Experimental models (non-human primate models, rodent models and zebrafish) are required to better characterize the cellular mechanisms and find biomarkers for diagnosis and/or therapy. In response to the increasing longevity of people, new alternative therapeutic approaches have attracted great interest worldwide, involving fibrates, connexins, neuroprotectants, photo/biomodulator agents and anti-inflammatory agents to inhibit the development and progression of insulted retinas [3]. Identifying the genetic, epigenetic and protein targets in retinal disorders might help to better focus the energy being applied to the development of new therapies in precision medicine. Of recent concern has been the Müller cell, which is one of the cell types suitable for this gene and protein analysis. Precision medicine encompasses the possibilities of developing selective/individualized approaches for patients non-responsive to the conventional therapy.

The purpose of this review is to present the current understanding of glial cells in physiological and pathological retinas, analyzing their structure, function and interactions with NGF and other factors, such as microenvironmental and epigenetic factors, focusing on the new findings about Müller cell reconditioning, to develop therapeutic approaches for clinical application in the near future.

## 2. Reactive Gliosis and the Multicellular Response

In vertebrates, the retinal glial cells, including microglia and macroglia (astrocytes and Müller cells), work in concert to guarantee local homeostasis [5]. Following injury, microglia, astrocytes and “reactive” Müller cells quickly respond to safeguard the entire retinal structure [6]. This activity is discussed below chiefly for Müller cells’ response, alone or in concert with astrocytes and microglia, and cell-to-mediator rules. Special attention has been devoted to NGF, TGFβ1 and VEGF.

### 2.1. Structure, Glial Cells and Homeostasis

Microglia, astrocytes and Müller cells are organized inside the retina from the choroidal towards the vitreous interface, interplaying with (I) rods and cones; (II) amacrine, horizontal cells; and (III) the ganglion cell layer (GCL), populated by retinal ganglion cells (RGCs) [7]. Microglial cells are primarily resident macrophage-like cell types (sentinels of the local innate immune system), populating the GCL until postnatal day 7 and thereafter spreading to the plexiform layers [8]. In adult retina, microglial cells are normally localized in both the Inner Plexiform and Outer Plexiform Layers (IPL and OPL) [9]. Upon retinal insult, microglial cells undergo a morphological transition to amoeboid-shaped cells (having retracted and bloated processes and modified cell surface marker expression) and migrate inside insulted regions for phagocytosis, trophic factor secretion and active remodeling of neurons/synapses [10]. The star-like retinal astrocytes are located in vascularized areas at the inner nuclear and plexiform retinal layers (INL and PRL), while they are absent from avascular zones [11]. Astrocytes’ residence strongly correlates with both the presence and distribution of retina blood vessels, exerting major roles in the development, maintenance and breakdown of the blood–retina barrier (BRB) of the inner retinal zone [12]. Müller cells are fully differentiated radial glial cells (90% of total glia) and, together with retinal neurons, take part in the “columnar micro-unit” (almost 10 × 10^6^ repetitive units per human eye), the smallest anatomical and functional structure required for visual signal transduction [13]. Inside each “micro-unit”, Müller cells exert different activities such as maintaining the extracellular space volume, facilitating ion/water homeostasis and buffering imbalances in the extracellular potassium (K^+^) and chloride (Cl^−^) ion concentrations via transcellular osmolyte currents [14]. Both K^+^ (Kir subfamily) and water (Aquaporin) channels on the Müller cell surface actively drive the process, allowing for perfect osmotic water transport (osmo-homeostasis) [15].

In retinogenesis, Müller cells are essential for i. providing a scaffold to allow oriented migration for post-mitotic young neurons; ii. allowing neurite outgrowth to create and maintain the neuroretinal architecture (regular formation processing) and iii. supporting neuronal survival [13,16]. In mature retina, Müller cells are located transversally to all nuclear and plexiform layers, with their endfeet contacting the vitreous cavity and their microvilli projecting into the subretinal space [17]. Müller cells allow light to guide to photoreceptors and buffer mechanical deformations of retinal tissue [18].

During their formation, the forming bodies project irregularly thick and thin processes onto the inner and outer limiting membranes [19]. The tight communication between Müller cell processes and retinal capillaries simplifies the crosstalk between the vasculature and neurons [20]. The neurotrophic/mechanical support provided by Müller cells and astrocytes encompasses i. energy to neurons, by upregulating nutrients and trophic/antioxidant factors (lactate/pyruvate) and removing metabolic waste (oxidative metabolism); ii. survival of photoreceptors/neurons; and iii. fast uptake/recycling of neurotransmitters and their precursors for correct neuronal signaling [13,17,21,22,23].

Any dysfunction of the lysosomal track belonging to glial cells, as a result of retinal insult and/or neurodegeneration and even the accumulation of non-degraded material, can evolve into impaired neuronal survival and functioning, as observed in selected in vitro studies [24,25].

### 2.2. Microenvironment: Cell-to-Cell and Cell-to-Mediator Considerations

The microenvironment is the outcome of cell-to-cell and cell-to-mediator interchanges and controls both resident and infiltrating cells, allowing for regulated crosstalk between neurons and accessory cells [26]. Appropriate neuronal signals (neurotransmitters and neurotrophins) preserve the glial population in a quiescent state, a requisite of extreme importance in the retinal network. As observed in experimental models of retina disorganization, such as reelin deprivation during retinogenesis, activated glial cells and particularly reactive Müller cells can provide prompt support to avoid neurodegeneration [13,27].

**Figure 2:**
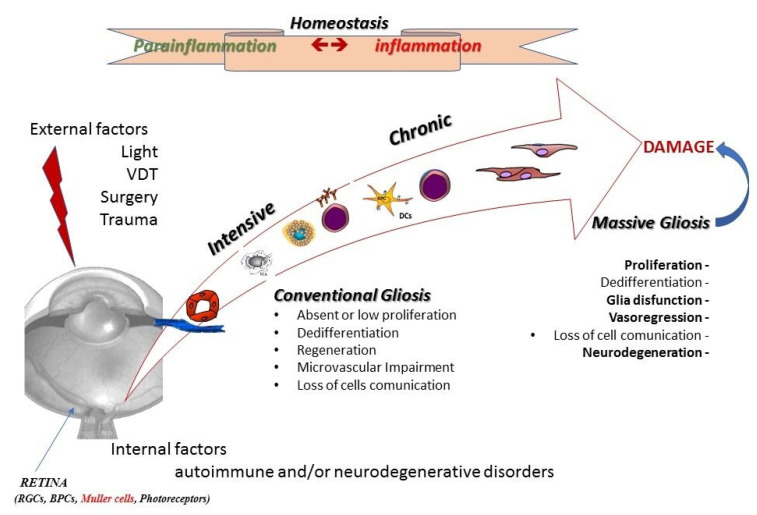
Overlapping acute to chronic cell-to-cell interplay: from conventional to reactive gliosis. The figure highlights the process from homeostasis to retinal damage, via an interplay of parainflammation toward inflammation—acute toward chronic insults—and neuroprotective toward degenerating overlapping activities, all including the switch from balanced to reactive gliosis.

Microenvironmental changes caused by traumas, infections, injuries, autoimmune diseases, iatrogenic factors, aging and even extracellular toxins can lead to retinal gliosis (Figure 2) [13,14]. Resulting morphological, biochemical and physiological changes occur to create a gliotic scar, which will have different features depending on the type/degree of injury, genetic/epigenetic background and selective inhibitory molecules from reactive glial cells, which might inhibit regular tissue repair in favor of abnormal repair/regeneration and later neurodegeneration [28,29].

By upregulating the expression of cytokines, chemokines and elements of the complement cascade in glial cells, activation compromises the integrity of the BRB and promotes a prompt protective response [30]. Sentinel microglia (macrophages), activated astrocytes and reactive Müller cells also release cytokines/chemokines, growth factors and matrix proteases (MMPs) to sustain the entire process of cell migration and differentiation, whilst RPE cells produce extracellular matrix and proinflammatory cytokines [30,31]. In recent years, different studies have focused on discriminating the biomolecular mechanisms that underlie changes in retinal cells between aging and well-established disorders [32]. Evidence from the study of mechanisms across inflammation, parainflammation and aging forms starting points for retinal diseases’ diagnosis and future alternative therapies [33].

## 3. Reactive Gliosis in Inflamed Retina: Reactive Müller Cells

**Figure 3:**
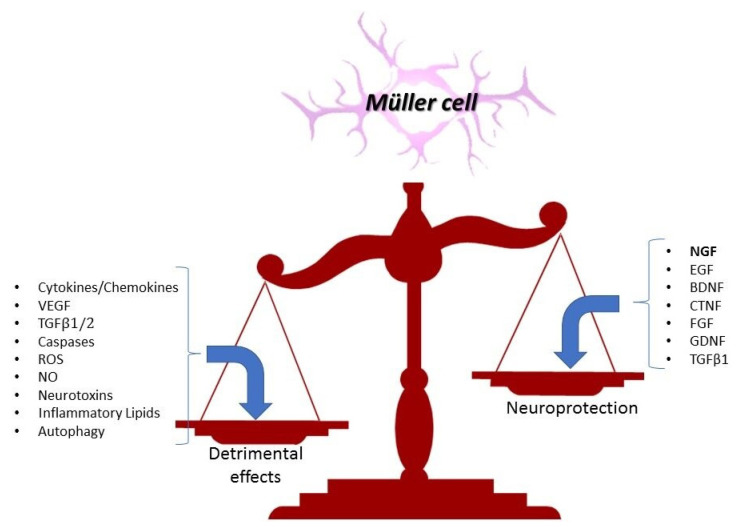
Reactive gliosis and Müller cells. The figure shows the different effects of Müller cells on neuroprotective to detrimental activities.

When exposed to noxious stimuli, astrocytes and Müller cells promptly converge to a “gliotic pathway” to guarantee retinal homeostasis, as directly observed in experimental models or indirectly by analyzing biological fluids (aqueous and/or vitreous) from AMD and DR patients undergoing intravitreal injections [1,16]. Figure 3 summarizes the involvement of glial cells as guardians in a healthy retinal microenvironment and their pivotal role in retinal disorders [34].

Microglia, astrocytes and Müller cells are active and/or partake in reactive gliosis, a process taking place at injured/inflamed retina (acute or chronic), including the degeneration and the modulation of cell phenotypes associated with the overt production of proinflammatory, profibrogenic and angiogenic mediators [30]. Either conservative or massive (non-neoplastic) proliferative gliosis can occur [35]. After the first insult, gliosis might evolve as a “conservative” or a non-proliferative and neuroprotective process, a cellular attempt to promote recovery, with restrictive effects on local damage and tissue remodeling [36]. Conservative gliosis is characterized by upregulation of GFAP (a specific marker of Müller glia activation); cellular hypertrophy; moderate or no decrease in K currents; and downregulation of glutamine synthetase (GS), cellular retinaldehyde-binding protein (CRALBP) and carbonic anhydrase (CA) [37]. While conservative gliosis could have physiological and well-controlled proliferative activity, massive and proliferative gliosis is characterized by a huge proliferation of Müller cells to form a “gliotic scar” [38]. Müller cells reenter the proliferation cycle, show strong Kir conductance downregulation and give rise to gliotic scars at both subretinal and epiretinal levels to fill spaces with a loss in neurons, pigment epithelium, blood vessels or photoreceptors [38]. In situ GFAP overexpression and/or increased immunoreactivity suggest a non-specific response of Müller cells to retinal injury and neurodegeneration, whilst being a specific marker (indicator) of Müller cell activation [39,40,41]. Under conditions of massive (proliferative) gliosis, regular glial–neuronal interactions are disrupted and insulted/impaired retina start to degenerate. Almost all retinal diseases are associated with reactive Müller cell-driven gliosis, either supporting the survival of retinal neurons (neuroprotective effects) or accelerating the progress of neuronal degeneration (detrimental effects) [16].

Impairments in the expression of GS, the enzyme involved in neurotransmitter recycling and glutamate detoxification, are a prominent example of a specific gliotic response [13]. The protective effect seems to involve the uptake of glutamate by the L-glutamate/L-aspartate transporter (GLAST), the primary glutamate transporter expressed by retinal astrocytes and Müller cells, postulated to contribute to glutamate clearance in order to protect RGCs from glutamate neurotoxicity [42]. Inside glial cells, glutamate is amidated by GS to form the non-neuroactive compound glutamine, quickly released from Müller cells and provided to neurons [13,14]. GS is exclusively expressed by Müller cells: in vitro cultured Müller cells protected RGCs against the excitotoxic effects of glutamate by increasing RGC survival [43]. Another protective Müller cell activity comprises the release of antioxidants [13,14]. Protection from oxidative and nitrosactive stress is achieved by the upregulation of antioxidants such as metallothioneins, lysozyme, the ferroxidase ceruloplasmin and hemeoxygenase and the release of reduced ascorbate [44].

### 3.1. Reactive Müller Cells in the Double Face of the Gliosis Process: Immunomodulation, Growth Factors, Microcirculation and Other Soluble Mediators

Inflammation, neuronal loss/damage and nerve degeneration can activate resident glia into effector cells (activated microglia/macrophages, reactive astrocytes and reactive Müller cells), with migration features and activation of effector genes [45]. Again, the neuroinflammatory response is supposed to be protective, while chronic neuroinflammation is considered highly harmful to the retinal environment, meaning its prompt inhibition may be a useful starting point in the development of appropriate treatments [46]. Reactive gliosis is involved in phagocytosis of exogenous substances, cell debris and extravasated serum proteins/hemoglobin, in order to clean the neuronal microenvironment of worsening products that might interfere with the mechanism of tissue repair [28]. As observed in several experimental models, microglia and/or Müller cells can actively phagocytose DNA fragments (developing retina) and/or dying cells in GCL/INL (postnatal retina) as well as dying photoreceptors that do not normally enter the ONL (in vivo models), and macrophages and Müller cells can phagocytose melanin granules in the subretinal space when experimentally implanted in animal models [47]. Interestingly, Müller cells can suppress both antigen- and IL2-driven proliferation of T-helper lymphocytes, in a cell-to-cell fashion, when T cells escape into the retina (animal model) and exert a primary inhibitory effect on T-cell proliferation (in vitro model) [48].

GFAP might also provide mechanical stability to an insulted retina [1]. In rabbits receiving sodium iodate, in the large areas missing RPE, photoreceptors were replaced with a subretinal scar consisting mainly of ascending processes of Müller cells [49,50]. Furthermore, experimental retinal holes produced in rabbit retinas were quickly filled with tissue consisting of Müller cell processes able to close the retinal holes [51].

In reactive gliosis, glial cells upregulate their production of neurotrophic factors to ensure the survival of neurons [52]. In in vitro studies, it was observed that photoreceptors and the inner retinal neurons survive thanks to direct/autocrine and indirect/paracrine (by means of Müller cells) release of neurotrophic factors [1,53]. Neuroprotective factors and cell locations are summarized in Table 1. Of interest, Müller cells allow for a prompt penetration of neurotrophic factors by producing/releasing MMPs with abilities to degrade the tight junction protein occludin [54].

In the epiretinal region, reactive Müller cells can transdifferentiate into contractile myofibrocytes that, in the presence of extensive/intense damage, continue to be activated even in the absence of stimulus (persistent gliosis), representing a bad prognostic indicator for retinal recovery [39]. Although DR is primary a microangiopathy, patients show early signs of altered neuroretinal function even before the appearance of microvascular lesions [55]. In a mouse model of oxygen-induced retinopathy (OIR), hypoxia caused strong GFAP induction and a GS reduction in Müller cells, both associated with increased TUNEL-positive cells (including neurons), implying neuronal degeneration [55].

The functional loss or even death of retinal neurons has been extensively attributed to uncontrolled reactive Müller cells, reactive gliosis (with intensive GFAP expression), induction of an acute-phase response and activation of inflammation-related genes [56]. As observed in proliferative retinopathies and thereafter proposed as a biomarker of DR progression, α2-macroglobulin (α2-MG) regulated ECM remodeling and cell migration through MMP-2 activation in Müller cells [57,58]. The observation of significant IL-1β upregulation in experimental diabetic retina pointed to IL-1β as a possible mediator of the acute-phase response raised by activated Müller cells [59].

In the inflammatory process, Müller cells are a major source of retinal IL-1β, the main cytokine triggering the neuroinflammatory cascade [60]. The retinal IL-1β-converting enzyme/caspase-1, known to produce biologically active IL-1β/IL-18, was found to be activated in both human and experimental DR, and elevated IL-1β levels were detected in diabetic mice and vitreous from diabetic patients [61]. High glucose triggers IL-1β secretion by retinal endothelial cells and upregulates IL-1β expression in Müller cells, in both autocrine and paracrine fashions [60]. IL-1β-exposed endothelial cells quickly progress to death, and IL-1β-exposed Müller cells express IL-6 through the activation of the p38 MAPK/NF-κB signaling pathway [62]. Dysregulated/persistent IL-6 production has been implicated in the development of autoimmune and chronic inflammatory-based retinal diseases (proliferative diabetic retinopathy) [63]. IL-1β was the lone cytokine able to stimulate proinflammatory IL-8 expression by Müller cells, mainly through the p38 MAPK and ERK1/2 pathways [64]. As a member of the CXC chemokine family, IL-8 works as a key activator/chemo-attractant for leukocytes and thereafter a crucial mediator of neovascularization [65]. Both increased IL-8 and IL-6 levels were quantified in aqueous and vitreous humors collected by patients with early and advanced stages of DR, including diabetic macular edema and proliferative DR [66].

### 3.2. Reactive Müller Cells in the Double Face of the Gliosis Process: Growth Factors

Müller cells are a good source of NGF and, in turn, NGF impacts Müller cell activity and suffering retina (DR, RP, etc.), which has been widely investigated in both experimental models and humans [67,68]. The early increase of NGF in injured/suffering retinal tissues has been associated with inflammatory/infiltrating cells as well as resident activated microglia, and particularly Müller cells, in addition to production from suffering neuronal subtypes (chiefly RGCs) [69,70].

Of relevance, early glial cell activation benefits the inflamed/insulted retina since activated glia promptly phagocytose apoptotic cells/debris/cytotoxins and secrete neurotrophic factors [71]. Unfortunately, Müller cells can perpetuate both vascular dysfunction and neurodegeneration by releasing inflammatory cytokines, VEGF and cytotoxic molecules [22,56]. Under particular stress conditions, reactive Müller cells (in concert with activated microglia) can strongly exacerbate neuronal cell dysfunction through the release of a wide range of proinflammatory agents toxic to neurons (merely RGCs), including cytokines/chemokines, glutamate, proteases and ROS/NO products, and also via elevated glucose levels [72].

In response to hypoxia, inflammation and glucose deprivation, Müller cells produce vessel-permeabilizing factors such as VEGF and TNF-α [73]. IL-1β and TNF-α cause an increased vesicular transport of serum proteins through vascular endothelial cells: serum-derived proteins accumulate in pericytes, perivascular microglia and Müller cells, suggesting the presence of a secondary barrier to extravasated serum proteins [74].

TNF-α can drive the upregulation of adhesion molecules, apoptotic cell markers and monocyte infiltration inside retina, contributing to the Müller cell dysfunction observed in DR [60]. In animal studies, TNF-α was reported to be involved in DR lesions, such as capillary degeneration, pericyte loss and increased retinal permeability [75]. Of interest, Müller cells exhibit a functional clock with a diurnal rhythm of Kir4.1, and increased TNF-α levels might be detrimental to the physiological rhythm and Kir4.1 expression [76]. In fact, high TNF-α levels lead to a severe decrease in Kir4.1 due to a reduced colocalization of Kir4.1 channels with synapse-associated protein (SAP97) and disorganization of the actin cytoskeleton [76].

As is widely reported, Müller cells release angiogenic and neurotrophic factors in an attempt to protect insulted retina [73]. Proangiogenic VEGF has been detected in vitreous and retinas of diabetic patients and animal models [77]. VEGF takes place in the majority of pathological changes observed in DR, by inducing the expression of inflammatory markers (TNFα, intercellular adhesion molecule-1 (ICAM-1)) and allowing the leucocytes to adhere to vessel walls [78]. In DR, VEGF can be primarily secreted by Müller cells under hyperglycemic conditions but also after prolonged exposure to fatty acids (oleic and linoleic acids) [79]. Chronic exposure to these factors sustains an increased vascular permeability, with further infiltration of stress-inducing factors and additional release of permeabilizing factors, perpetuating the mechanism and resulting in a deleterious positive feedback cycle. In spite of the fact that low levels of VEGF promote the survival of vascular endothelial cells and retinal neurons, excessive levels of VEGF may contribute to greater progression of retinal diseases by inducing vascular leakage and neovascularization [80]. High concentrations of VEGF cause endothelial cell hyperplasia, resulting in capillary nonperfusion and other vascular alterations typifying DR [81]. Massive gliosis includes the proliferation and migration of Müller cells, with the original purpose of assembling new connections with the remaining nerve cells [1]. Although the mechanisms responsible for glial proliferation are not completely known, the X-linked Inhibitor for Apoptosis Protein (XIAP) may be responsible for increased proliferation in Müller cells [82]. In has been reported that high-glucose conditions dysregulate XIAP/apoptosis signaling, reducing apoptosis and enhancing the survival/proliferation of Müller cells [82]. XIAP expression strongly correlated with VEGF expression under high-glucose conditions, while XIAP inhibition (by embelin, a small molecule that specifically inhibits XIAP) downregulated VEGF expression, suggesting a regulatory role of XIAP in glucose-induced VEGF expression [83]. XIAP participates in the regulation of VEGF expression, being a crucial mediator of several proteins/factors (NFkB, p65, p38, TNF-α and IL-1β, uPA, CREB, ERβ, Stat3 and HCAM) [84]. In turn, VEGF was found to enhance XIAP expression, suggesting a possible positive feedback loop to further increase VEGF production and promote Müller cell proliferation [83,84].

Despite the well-known growth factor neuroprotective actions, Insulin-like Growth Factor-1 (IGF-1) is critically involved in retinal neovascularization: chronic elevated levels of IGF-1 can lead to neurodegeneration with neuronal loss, electroretinography impairment and increased cleaved caspase-3-positive cells [85]. In proliferative DR, IGF-1 can contribute to the process of increased VEGF and neovascularization [86]. Under hypoxic conditions, IGF-I-stimulated HIF-1 activity in Müller cells through PI-3-kinase and MAPK signaling pathways leads to VEGF mRNA expression [87]. IGF-1 also plays a pivotal role in ECM remodeling by influencing MMP release by activated Müller cells [88]. In PDR and proliferative vitreoretinopathy (PVR), migration and proliferation of Müller cells were also associated with ECM degradation by means of MMPs [89]. Müller cells express MMP-2 (gelatinase A) and MMP-9 (gelatinase B), implicated in matrix degradation and cell migration [90]. As observed in cultured Müller cells, the exposure to TNF-β upregulates MMP-9 expression, whereas exposure to α2M induces MMP-2 activation and regulates membrane type 1 MMP (MT1-MMP) activity [91]. IGF-1-exposed Müller cells induce MMP-2 production, facilitating cell migration and repopulation in the damaged host retina [92]. Moreover, the IGF-1/IGF-1R system regulates active MMP-2 levels in Müller cells, contributing to ECM remodeling during retinal neovascular processes [92]. Finally, MMPs impair the tight junction function in retinal endothelial and pigment epithelial cells, by proteolytic degradation of the tight junction protein occludin, promoting vascular leakage with well-known deleterious effects on retinal homeostasis [93].

### 3.3. Reactive Müller Cells in the Double Face of the Gliosis Process: Microcirculation and Other Soluble Mediators

Corroborating data sustain that reactive Müller cells may contribute to retinal damage not only directly by the release of toxic molecules but also indirectly by an impairment of neuron-supportive functions. As introduced above, the major physiological function of Müller cells is the regulation of extracellular glutamate through the high-affinity glutamate transporter GLAST (Glutamate Aspartate Transporter, the human analog is named EAAT1) [94]. In vertebrate retina, GLAST is exclusively expressed by Müller cells and astrocytes, works on the removal of glutamate from the synaptic gap and removes glutamate signaling and protection of neurons from glutamate’s excitotoxic actions [94]. Hyperglycemic conditions in cultured Müller cells stimulate the expression of NOS and cyclooxygenase-2 (COX2) with a subsequent increase in NO and production of cytotoxic PGs triggering glucose-mediated oxidative stress [95]. Hyperglycemia-induced oxidative stress can cause the dysfunction of GLAST molecules in Müller cells. In rat Müller cells, the GLAST function significantly decreased after just 4 weeks from streptozotocin-induced diabetes [96]. Furthermore, the activity and content of GS were significantly reduced in diabetic retina, with a subsequent decrease in the conversion of glutamate to glutamine necessary for neurotransmitter regeneration [97]. These observations are consistent with reports showing an early and significant increase in glutamate in retinas from experimental diabetes and in vitreous from DR patients, suggesting that DR may be associated with glutamate-induced excitotoxicity [98]. On the other hand, the oxidative stress dysfunction of Müller cell GLAST, resulting in increased glutamate levels, may create a positive feedback loop that further increases oxidative stress, sustaining diabetic complications in retina [22]. In failing to preserve glutamate homeostasis in diabetic retina, Müller cells may contribute to increased oxidative stress by promoting an i. increase in polyol synthesis, ii. formation of advanced glycation end products, iii. activation of protein kinase C and iv. enhanced flux through the hexosamine pathway, all molecular mechanisms implicated in the pathogenesis of diabetic complications [22,99,100]. Müller cells also lose their ability to regulate glutamate homeostasis in glaucoma-associated retinopathy [51]. Due to a reduction in GLAST biosynthesis, glutamate accumulates in intercellular space, inducing neuronal death [101].

In a rat model of Branch Retinal Vein Occlusion (BRVO), VEGF expression was upregulated as soon as 1 day from BRVO and balanced after 3 days, by means of PEDF upregulation in the neuroretina and retinal pigment epithelium [102]. This fast VEGF upregulation contributed to the breakdown of the inner BRB, resulting in the formation of cystoid spaces around retinal vessels [103]. After BRVO, Müller cells display downregulation/inactivation of Kir4.1 channels, with decreased potassium currents causing uncoupling of Aquaporin 4-mediated water transport from K^+^ currents, resulting in altered transglial water transport as well as impaired fluid absorption from retinal tissue (edema formation) [104]. In a pig model of acute BRVO, neural cell death occurs early due to inflammation and breakdown of osmohomeostasis, as shown by GFAP and IL-8 upregulation and Kir 4.1 downregulation, all aspects associated with gliotic changes in Müller cells [1,22]. For the progression of DR, a double contribution to glutamate toxicity was observed for Müller cells: a decreasing glutamate uptake (direct effect) and a decreasing K^+^ uptake (indirect effect). K^+^ conductance of Müller cells was reduced in experimental diabetes as well as in patients with proliferative DR [22]. A downregulation of K^+^ currents associated with an alteration in the expression pattern of the major K^+^ channel of Müller cells (Kir4.1), normally present in perivascular and endfeet membranes of the cells, was observed in diabetic rat retinas [51]. The downregulation in Kir 4.1 channels, which mediate K^+^ efflux to the blood, seems to be the main reason for hydroelectrolytic imbalance, which, in turn, may contribute to retinal degeneration and accumulation of fluid in the retinal tissue [51]. Therefore, osmotic swelling of Müller cells and their subsequent dysfunction may contribute to the development of retinal edema [105]. The mislocalization of Kir 4.1 channels reduces K currents in retinal cells, altering synaptic activity and threatening cell survival [104]. Similarly, changes in the distribution of the water-selective channels (AQP1 and AQP4) lead to an increased number of apoptotic neurons, thereby contributing to retinal dysfunction [106]. AQP4 is a water channel protein showing a similar polarized distribution of Kir4.1 at the endfoot membranes of Müller cells; AQP4 facilitates water flux at the glio-vascular and glio-vitreal interfaces [107]. Under pathological conditions, AQP4 expression has been shown to be largely unaltered or even increased, whereas the expression of Kir4.1 is decreased [66]. This may result in uncoupling of transglial K^+^ and water transport, which may contribute to retinal degeneration and edema [14]. In diabetic rat retina, high glucose triggers NO with increased AQP4 expression in Müller cells, suggesting NO as a possible inducer of diabetic retinal edema associated with Müller cell swelling [14,51].

## 4. Old and Recent Findings to Support Future Precision and/or Individualized Medicine

Individuals differ in their genetic and protein makeup and might require different therapeutic approaches. This points to a need for precision, personalized or even individualized medicine, which appear highly suitable to provide appropriate solutions to certain complex disorders. According to the definition promulgated by the National Institutes of Health (NIH), precision medicine refers to a new treatment and prevention method based on understanding of individual genes, the environment and lifestyle [108]. Nevertheless, the word “personalized” could be misinterpreted as implying that treatments and preventions are being developed uniquely for each individual; instead, precision medicine is a healthcare model that focuses on identifying which approaches will be effective for which patients based on genetic, environmental and lifestyle factors [109]. The National Research Council highlighted that the terms “precision medicine” and “personalized medicine” have overlapping meanings used “interchangeably” [108].

Personalized medicine is a healthcare model that focusses on individual tailoring on the basis of a person’s genes, lifestyle and environment (customized therapy) [110]. Precision medicine, by applying genomics, proteomics and other relevant technologies to analyze and identify the biomarkers of large sample groups and specific diseases, seeks to provide precise and individualized treatment to certain patients and for certain diseases [111]. By combining knowledge on the human body, ethics, economics, sociology and other knowledge elements, precision medicine aims to minimize iatrogenic damage and medical expenses in order to achieve an optimal therapeutic effect [112]. Precision medicine has widely entered the ocular field, with attempts to provide solutions to disorders producing hypovision or complete vision loss. Gaining an understanding of soluble factors able to contribute to pathological and clinical stages leading to vision loss in aged retinas as well as in autoimmune-linked retinopathy (DR) represents a step forward for developing new therapeutic tools (precision medicine).

Changes in the biochemical pathways and how soluble factors or even overexpressed receptors promote pathophysiological developments (proinflammatory cytokines/chemokines and adhesion molecules) offer key information on how we may counteract local inflammation-mediated leukostasis, retinal ischemia and neovascularization [113].

### 4.1. Necessity of Soluble Targets: Traditional Experimental Models and Others of Recent Attention

Considering that the majority of these soluble mediators are released by Müller cells and quantified in vitreous and/or vitreal reflux, depending on the state of disease and intervention, it is reasonable to identify Müller cells as the main suppliers of the overall in vivo synthesis [114,115]. Levels of inflammatory cytokines IL-1β, IL-6, IL-8, IL-17A and TNF-α in aqueous and vitreous humors have been associated with different levels of pathogenesis (severity) and proposed as prognostic and/or likewise preventive targets of DR [116]. Growth factors, cytokines/chemokines, oxidative metabolites and matrix-degrading enzymes (proteases, metalloproteinases (MMPs) and their tissue-specific inhibitors (TIMPs)) have been quantified in vitreous (including non-invasive vitreal reflux sampling) and subretinal fluids in humans and retinal tissues from animal models [117]. Vujosevic and collaborators characterized and compared the protein profiles in aqueous humors collected from patients undergoing cataract surgery, grouped as no-DR and with DR in the presence or absence of retinal edema [118]. This comparison highlighted the presence of some inflammatory mediators and particularly pointed to some mediators of Müller cells’ derivation in no-DR vs. DR aqueous humors, with interesting predictive effects [118]. A recent study tested the use of α-aminoadipic acid (a specific glio-toxic inducer) to block gliosis through selective obstruction of glutamate uptake, contributing to the reduced neurodegeneration observed in glaucoma [119].

### 4.2. Target Therapy: Beyond the Conventional Approaches

Müller cells are found in all vertebrates and react to injury by entering reactive gliosis, inhibiting the regeneration process [120]. The major cause of untreatable blindness worldwide is retinitis pigmentosa (RP), due to the death of photoreceptors and/or RGCs [121]. Since RP is caused by more than 3000 mutations in more than 50 different genes, diverse animal models of RP have been produced as a good starting point, from the RCS rat to the Pde6brd10/J (rd10) mouse [121]. Studies on these experimental models, including those on gene, cell and regenerative therapies, as well as pharmacological treatments, are gradually progressing or have the potential to lead to clinical trials [122,123].

Several research groups have investigated gene and microRNA (miRNA) expression in the retina, comparing it to other tissues and classifying different miRNAs in retina [124,125].

The potential of intravitreal delivery, of molecular-based agents is discussed below. Chiefly, gene (ocular) therapy replaces or adds a gene that causes a specific disease with a healthy copy or “knocks out” (inactivates) an aberrant gene through the delivery of therapeutic agents into the eye in a localized and sustained fashion [125]. Since Müller cells are involved in multiple pathological retinal diseases and produce/release different signaling molecules influencing retinal neuronal activity, survival and repair, these cells appear to be potential targets for gene therapy in the treatment of retinal degeneration and likewise in the prevention of overt Müller cell activation [1]. In 1998, Sakamoto and co-workers demonstrated for the first time that following intravitreal injection of adeno-associated viruses (AAVs), the gene-transfected cells were located in the GCL and were positive for vimentin, GFAP and S100 protein antibodies, suggesting that these cells were primarily Müller cells and confirming the convenience of using AAV for gene therapy [126]. Sakamoto et al. designed AAV-based gene therapy that preferentially targets RPE and Müller cells [126]. Today, adenoviral vector intravitreal injection after surgery is considered an efficient and safe method for transferring genes to neural retinal cells [127]. The possibility to knock down Müller cells bearing a derived VEGFA-splicing variant (VEGF164) was described by Jiang and coworkers to provide a safe intravitreal approach to inhibiting VEGF164 and thereafter reducing/counteracting neovascularization [128]. In line with this, Rhee and co-workers provided evidence for targeting Müller glia and promoting the protection/survival of photoreceptors by means of exogenous hCNTF, which relies on initial signaling through the gp130 receptor in Müller cells and cytokine induction through gp130 in photoreceptors [129]. Byrne and co-workers evaluated the structural and functional retinal rescue (Rs1h−/− mouse transport) following intravitreal injections of three different viral vectors targeting different subsets of retinal cells (including photoreceptors and Müller cells) [130]. Given their implication in RS1 gene delivery and structural/functional support to retinal neurons, Müller cells appear to be strong candidates for gene therapy, due to their morphology, location and relative preservation in late-stage retinal degeneration [131]. Very recently, He and coworkers reported that Müller cells could de-differentiate into potential retinal stem cells, as observed in studies with miR-124 in axon growth of ganglion cells derived from Müller cells both in vitro and in vivo [132].

Gene and protein expression are significantly altered in aging, although the pathway of this altered expression is still not clear [133]. A dysregulation in parainflammation associated with increasing age has also been described in retinas [134]. Genetic susceptibility is a possible cause of the development and progression of several retinal disorders mainly associated with aging (AMD and DR) [135]. Epigenetic markers, including DNA methylation, histon acetylation/deacetylation and target gene expression, have been reported so far [136]. Different miRNA profiles characterize developing, adult and pathological retinas, chiefly miRNAs involved in cell proliferation, apoptosis and even malignant transformation [125,137]. MiRNAs are small (18 to 24 nucleotides), highly conserved and noncoding molecules that regulate gene expression in a wide variety of tissues and cell types, including retinas and retinal cells [138]. Several groups have begun exploring the expression and function of miRNAs in Müller cells [139]. In a recent study of oxidative-stress-related retinopathy in Müller cells, a small RNA library from the glyoxal-treated rat Müller cell line (glyoxal-treated rMC-1) was produced [140]. In mammalian retina, Müller cells predominantly respond to injury by undergoing non-proliferative gliosis [140]. Contrary to zebrafish, Medaka Müller glia (olMG) cells work like progenitors and exhibit a restricted capacity to regenerate the retina [141]. After injury, olMG cells proliferate but fail to self-renew and ultimately only restore photoreceptors [141]. As an explanation, proliferating olMG cells do not maintain sox2 expression, as zebrafish do, and sustained sox2 expression in olMG cells confers regenerative responses similar to those of zebrafish MG (drMG) cells [141]. In chick retinas, NMDA exposure induced Müller glia to re-enter the cell cycle, dedifferentiate, express genes common to embryonic retinal progenitors and produce some amacrine, bipolar and glia cells [142]. Consequently, other than mammals, it has so far been determined only that adult zebrafish regenerate all retinal neurons through the activation, dedifferentiation and proliferation of Müller glia [141]. Since mammalian Müller cells are unable to regenerate retinal neurons, it may be useful to look for factors allowing zebrafish retinal tissue to regenerate and to transfer this information to human retinas. Zebrafish are small freshwater teleosts appreciated for their use in disease modeling, particularly for neurodegeneration and metabolic disease, and they appear a suitable animal model for retinal degeneration [143].

Forthcoming cell-based therapy should be mentioned since cell culture models can provide useful information from the study of the development and functional activities of cells under pathological conditions. Although a 1D cell model cannot reproduce the behavior of cells inside a lesioned tissue, its 2D or 3D counterparts have recently attracted interest for this reason [144]. Phillips et al. showed that immortalized Müller cells can display some characteristics of retinal stem cells and progenitors [144,145]. The demonstration that these stem cells when exposed to specific growth factors (FGF2, EGF) can produce spheres (neural stem cells, retinal progenitors and Müller glia) implies the possibility of building a retinal-like environment [144,145,146]. Although a hallmark of dedifferentiation, the proliferation of Müller glia, and particularly Müller cells, as well as their profiling with respect to transcription factors and cell cycle regulators, might be essential for mimicking in vitro the function of retinal progenitors (latent neurogenic capacity) for retinal regeneration [147]. Unfortunately, proliferation of Müller glia is often associated with gliogenic rather than neurogenic tasks (glial scar formation), implying that the lone stimulation of Müller glial proliferation might not be sufficient to enhance the neurogenic potential of mammalian retinas. For this reason, gaining an understanding of the cellular linkage between lineage-specific transcription factor expression and cell cycle progression is essential to developing accurate strategies for triggering the regeneration of mammalian retinas [147]. Both the NGF upregulation and Notch1 downregulation observed in the transient proliferation, dedifferentiation and neurogenesis of in vitro cultured Müller cells and the mimicking of acute retinal injury provided a profiling of molecules in charge of the regenerative potential of Müller cells [148]. As they are known to regulate a variety of processes during the development and regeneration of retinas, the above-mentioned targets and the translational repressor miRNAs might open up alternative routes for Müller glia and likewise Müller cell activation/function, offering therapeutic targets that may aid in overcoming retinal gliosis [149].

## 5. Conclusions

Taken together, gliosis is a multicellular response, encompassing the crosstalk between Müller cells, astrocytes, microglia and infiltrating cells belonging to the innate and immune systems, which creates severe impairments in suffering retina [1]. Cell-to-cell and cell-to-mediator crosstalk can influence neurons and accessory cells, and the initial trigger may be neuronal or synaptic damage or even death secondary to intrinsic neuronal changes [94]. Müller cells acquire a reactive phenotype in many retinal diseases, such as retinal detachment, retinal degeneration and glaucoma, as a response to the loss of retinal neurons. This protective phenotype (reactive Müller cells) was introduced several decades ago when Willbold and Layer postulated that Müller cells perform several functions in the complex architecture (axonal fiber guidance and synapse stabilization) and homeostasis of the retina, as tested in vivo and in vitro, and subsequently it is improved [150]. But activated Müller cella and reactive gliosis can display both (neuro) protective and toxic (detrimental) effects in many eye conditions and on retinal neurons. The gliotic response may exert biphasic effects, depending on the time or amplitude, and dysregulation or overstimulation of protective glial responses may also result in detrimental effects [28]. Useful ways to counteract abnormal tissue and/or cell activity in the retinas and provide alternatives to guarantee neuroprotection and/or prevention of degeneration are the intravitreal delivery of humanized antibodies, non-toxic or selectively toxic agents and miRNAs. Precision therapy will deliver these novel treatment approaches, resulting in different levels of functioning [151].

The above-reported findings on reactive Müller cells and the latest-generation methods highlight the importance of a rational and focused approach to gene therapies and evaluation of the strategies used for viral-vector-mediated delivery, including the cell type targeted, a minimally invasive delivery method and a long-lasting gene therapy.

## Figures and Tables

**Table 1:** Neuroprotective factors.

Target	Action	Cells
*NTs*	(Neurotrophin-3 and -4) survival or apoptotic effects	All retinal cells
*BDNF*	Photoreceptor survival	Müller cell production enhances photoreceptor survival
*NGF*	Survival	All retinal cells
*VEGF*	Survival	VEGF supports the survival of endothelial cells, retinal neurons and glial cell proliferation, neuroprotection and neurogenesis
*bFGF*	Survival	All retinal cells
*Osteopontin*	Survival	RGCs
*BCL2*	Survival	RGCs

## Data Availability

Not applicable.
